# Multi-level suppression of receptor-PI3K-mTORC1 by fatty acid synthase inhibitors is crucial for their efficacy against ovarian cancer cells

**DOI:** 10.18632/oncotarget.14591

**Published:** 2017-01-10

**Authors:** Renate Wagner, Gerald Stübiger, Daniel Veigel, Michael Wuczkowski, Peter Lanzerstorfer, Julian Weghuber, Emmanouil Karteris, Karin Nowikovsky, Nastasia Wilfinger-Lutz, Christian F. Singer, Ramón Colomer, Bellinda Benhamú, María Luz López-Rodríguez, Peter Valent, Thomas W. Grunt

**Affiliations:** ^1^ Division of Oncology, Department of Medicine I, Medical University Vienna, Vienna, Austria; ^2^ Comprehensive Cancer Center, Medical University Vienna, Vienna, Austria; ^3^ Department of Biomedical Imaging and Image-guided Therapy, Medical University Vienna, Vienna, Austria; ^4^ Center for Biomarker Research in Medicine, Graz, Austria; ^5^ University of Applied Sciences Upper Austria, School of Engineering and Environmental Sciences, Wels, Austria; ^6^ Department of Biosciences, School of Health Sciences and Social Care, Brunel University London, Uxbridge, UK; ^7^ Department of Obstetrics/Gynecology, Medical University Vienna, Vienna, Austria; ^8^ Department of Medical Oncology, Hospital Universitario La Princesa and Spanish National Cancer Research Center (CNIO), Clinical Research Program, Madrid, Spain; ^9^ Departamento de Química Orgánica I, Facultad de Ciencias Químicas, Universidad Complutense de Madrid, Madrid, Spain; ^10^ Division of Hematology and Hemostaseology, Department of Medicine I, Medical University Vienna, Vienna, Austria; ^11^ Ludwig Boltzmann Cluster Oncology, Medical University Vienna, Vienna, Austria

**Keywords:** AMPK, fatty acid synthase (FASN), lipids, mTORC1, REDD1

## Abstract

Receptor-PI3K-mTORC1 signaling and fatty acid synthase (FASN)-regulated lipid biosynthesis harbor numerous drug targets and are molecularly connected. We hypothesize that unraveling the mechanisms of pathway cross-talk will be useful for designing novel co-targeting strategies for ovarian cancer (OC). The impact of receptor-PI3K-mTORC1 onto FASN is already well-characterized. However, reverse actions–from FASN towards receptor-PI3K-mTORC1–are still elusive. We show that FASN-blockade impairs receptor-PI3K-mTORC1 signaling at multiple levels. Thin-layer chromatography and MALDI-MS/MS reveals that FASN-inhibitors (C75, G28UCM) augment polyunsaturated fatty acids and diminish signaling lipids diacylglycerol (DAG) and phosphatidylinositol 3,4,5-trisphosphate (PIP3) in OC cells (SKOV3, OVCAR-3, A2780, HOC-7). Western blotting and micropatterning demonstrate that FASN-blockers impair phosphorylation/expression of EGF-receptor/ERBB/HER and decrease GRB2–EGF-receptor recruitment leading to PI3K-AKT suppression. FASN-inhibitors activate stress response-genes HIF-1α-REDD1 (RTP801/DIG2/DDIT4) and AMPKα causing mTORC1- and S6-repression. We conclude that FASN-inhibitor-mediated blockade of receptor-PI3K-mTORC1 occurs due to a number of distinct but cooperating processes. Moreover, decrease of PI3K-mTORC1 abolishes cross-repression of MEK-ERK causing ERK activation. Consequently, the MEK-inhibitor selumetinib/AZD6244, in contrast to the PI3K/mTOR-inhibitor dactolisib/NVP-BEZ235, increases growth inhibition when given together with a FASN-blocker. We are the first to provide deep insight on how FASN-inhibition blocks ERBB-PI3K-mTORC1 activity at multiple molecular levels. Moreover, our data encourage therapeutic approaches using FASN-antagonists together with MEK-ERK-inhibitors.

## INTRODUCTION

Ovarian cancer (OC) represents the most lethal malignancy of the female reproductive system [1–3]. Although new anticancer drugs have significantly improved the five-year survival of OC, overall mortality yet remains invariably high [4]. Thus, a deeper understanding of the molecular background of OC is required. Many OCs harbor hyperactive oncogenic drivers in the ERBB-phosphatidylinositol 3-kinase(PI3K)-mechanistic target of rapamycin complex 1 (mTORC1) signaling pathway. They exhibit ‘PI3Kness’ and are sensitive to drugs that target this pathway [5, 6]. ERBB-PI3K-mTORC1 controls diverse cellular functions including proliferation, survival, growth, migration, autophagy, angiogenesis, metabolism, and energy balance [7]. At the molecular level, ERBB membrane receptors activate PI3K, which converts phosphatidylinositol 4,5-bisphosphate (PIP2) to phosphatidylinositol 3,4,5-trisphosphate (PIP3). PIP3 stimulates AKT, which then inactivates TSC2 [8]. Thereby, the TSC1/2 tumor suppressor complex loses its ability to block RHEB–the upstream activator of mTORC1–and enables mTORC1 downstream signaling. S6K1 and 4EBP1 are the main targets of mTORC1 and control cell division and protein biosynthesis. TSC1/2 functions as a regulator of cell homeostasis. It receives activating inputs from REDD1 (DDIT4, RTP801) and AMPK–two sensors of nutritive and energetic shortage, which counteract AKT-mediated induction of mTORC1 and block cell proliferation.

Cancer cells require large amounts of proteins, nucleotides, lipids and energy for growth. In recent years, the reprogramming occurring in cancer cell metabolic networks has gained considerable attention. Research in this field will likely generate new concepts for improved anticancer treatment. For instance, lipid biosynthesis has been found to be hyperactive in most malignant tissues. Hence, fatty acid synthase (FASN), the enzyme responsible for *de novo* lipogenesis, is overexpressed in tumors including OC and is considered a useful tumor marker. It signifies unfavorable outcome and represents a hallmark of cancer [9–12]. At the biochemical level, acetyl-CoA is generated from citrate and is further processed to malonyl-CoA. Both CoA-conjugates are used by FASN to form the saturated long-chain fatty acid palmitic acid (PA; 16 : 0) [10]. Blockade of FASN has been demonstrated to exert anticancer effects in human OC [11] and thus represents an appealing strategy for treatment.

Available data suggest that ERBB-PI3K-mTORC1 up-regulates FASN by induction of the transcription factor SREBP-1c [13]. We recently demonstrated that FASN in turn can stimulate PI3K-mTORC1 signaling and contrariwise blockade of FASN impairs PI3K-mTORC1 [14, 15]. However, the mechanisms of this inhibitory action from FASN onto ERBB-PI3K-mTORC1 remain elusive. Here we demonstrate that blockade of FASN activates the mTORC1 repressors REDD1 and AMPKα causing mTORC1 downstream inhibition. This is accompanied by compensatory MAPK ERK activation. Accordingly, combination of' FASN-blockers with MAPK pathway inhibitors yields stronger growth inhibition than single FASN-inhibitor treatment. Herewith, we provide the first in-depth analysis on how FASN-inhibition blocks ERBB-PI3K-mTORC1 activity at various molecular levels.

## RESULTS

### OC cell lines reveal diverse sensitivities against FASN-inhibitors

We and others have shown that FASN-inhibitor sensitivities and FASN protein expression levels correlate with each other, while differing markedly between individual OC cell lines [12–16]. Thus, the IC_50_ values for growth inhibition after 72 h exposure to the prototypic FASN-inhibitor C75 or to the more advanced compound G28UCM vary considerably in the cell lines used (IC_50_ of C75: HOC-7 = 29 ± 1 μM, SKOV3 = 27 ± 5 μM, OVCAR-3 = 18 ± 3 μM, A2780 = 22 ± 5 μM; IC_50_ of G28UCM: HOC-7 = 21 ± 1 μM, SKOV3 = 10 ± 3 μM, OVCAR-3 = 4 ± 1 μM, A2780 = 3 ± 1 μM) (Supplementary Figure 1). Therefore, isoeffective instead of identical drug concentrations have to be used for comparison of FASN-inhibitor effects in different cell lines. For instance, 72 h of exposure to 40, 25, 20 or 10 μM G28UCM, or to 40, 35, 20 or 30 μM C75 yield roughly similar growth inhibition (60–70 %) in SKOV3, HOC-7, OVCAR-3 or A2780 cells, respectively.

### FASN-inhibitors down-regulate oleic acid (OA), diacylglycerol (DAG) and phosphatidylinositol 3,4,5-trisphosphate (PIP3), but elevate polyunsaturated fatty acids (PUFA) and malonyl-CoA

**Figure 1:**
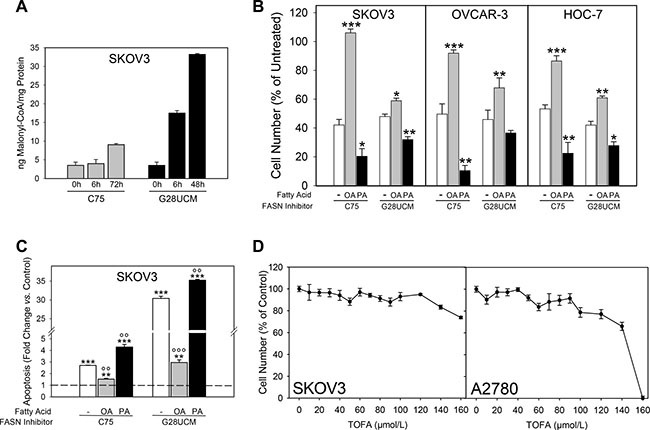
The degrees of accumulation of malonyl-CoA and depletion of oleic acid (OA) upon inhibition of fatty acid synthase (FASN) in ovarian cancer (OC) cells depend on the particular inhibitors used (**A**) Malonyl-CoA is quickly and strongly accumulated by G28UCM, but much less by C75. (**B**) Supplementation of OA, unlike PA, antagonizes C75-mediated growth inhibition more efficiently than G28UCM-mediated growth inhibition. Data obtained after exposure to C75 (80 μM for SKOV3 and HOC-7, 40 μM for OVCAR-3) or G28UCM (80 μM for SKOV3, 15 μM for OVCAR-3, 30 μM for HOC-7) ± 70 μM OA or PA are presented. 1.5 × 103 (SKOV3, OVCAR-3) or 0.5 × 103 (HOC-7) cells/well were seeded in a 96 well plate and treated for 72 h. Note: In these experiments cultures contained bovine serum albumin (for details of dissolving fatty acids see Materials and methods), which lowers the efficacy of FASN-inhibitors [38]. Therefore, higher concentrations of C75 and G28UCM had to be applied in order to achieve marked inhibition of cell growth. Data from untreated controls (not shown in chart) were set to 100% and those from treated cultures were related to it. Means ± SD, n = 3. *p < 0.05, **p < 0.01, ***p < 0.001 FASN-inhibitor alone vs. FASN-inhibitor combined with OA or PA. One-way ANOVA and Scheffe-test. (**C**) C75 is a weak inducer of apoptosis compared to G28UCM. Moreover, OA fully abrogates C75-induced cell death, but only partially inhibits G28UCM-mediated apoptosis. Means ± SD, n = 3. **p < 0.01, ***p < 0.001 treated vs. untreated control (dashed horizontal line at y = 1). °°p < 0.01, °°°p < 0.001 FASN-inhibitor alone vs. FASN-inhibitor combined with OA or PA. One-way ANOVA and Scheffe-test. (**D**) Growth inhibition of OC cells after 72 h exposure to TOFA, an inhibitor of acetyl-CoA carboxylase.

Acetyl-CoA carboxylase converts acetyl-CoA to malonyl-CoA. Both intermediates are used by FASN to generate the saturated fatty acid (FA) palmitic acid (PA (16 : 0)), which is the source for most other lipids including monounsaturated FA (MUFA) oleic acid (OA (18 : 1(9Z))). Blockade of FASN therefore leads to loss of FAs and to accumulation of malonyl-CoA (Figure 1A). Both conditions can be harmful to the cells [17]. We demonstrate that addition of exogenous OA, unlike PA, partially abolishes FASN-inhibitor-mediated growth arrest and apoptosis (Figure 1B, 1C). Inhibitors of acetyl-CoA carboxylase such as TOFA, on the other hand, induce FA deficiency without accumulation of malonyl-CoA and impair OC cell growth only at very high concentrations (Figure 1D). These data suggest that cytotoxicity of FASN-blockers is most likely mediated by both OA deprivation and malonyl-CoA accumulation.

**Figure 2:**
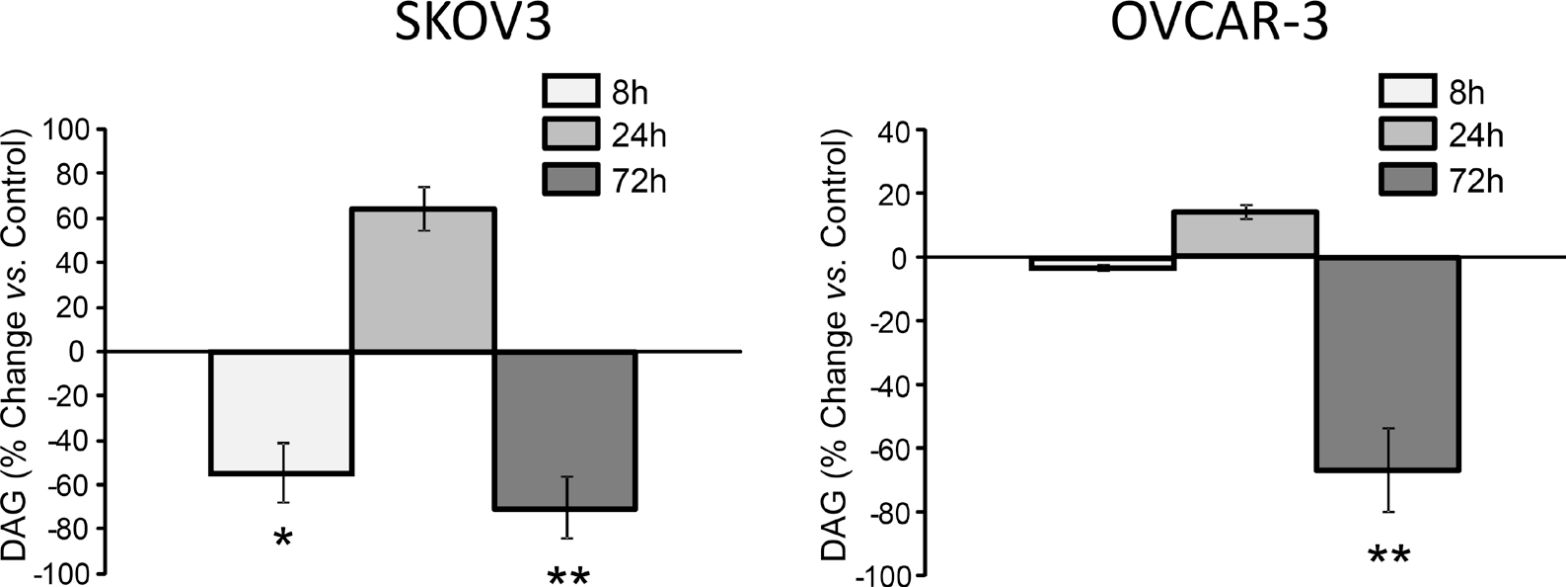
Large fluctuations of DAG upon inhibition of FASN by G28UCM as demonstrated by thin-layer chromatography in SKOV3 (left) and OVCAR-3 (right) Values represent % change of total DAG in treated cells relative to untreated control cells (horizontal 0-line). Means ± SD, n = 3. *p < 0.05, **p < 0.01 vs. control cells, one-way ANOVA and Scheffe-test.

Next we applied thin-layer chromatography for mapping the effects of FASN-inhibition on the major lipid classes. DAG is formed by cleavage of phosphatidylinositol 4,5-bisphosphate (PIP2) [18] and transfers signals from membrane receptors to protein kinase C (PKC). DAG is also a precursor of membrane glycerophospholipids and plays a key role in *de novo* lipogenesis and lipid remodeling in cancer [19]. Interestingly, DAG levels are decreased after 8 h of exposure to G28UCM (−54% in SKOV3, −5% in OVCAR-3). This is followed by a transient rebound at 24 h (+65% in SKOV3, +15% in OVCAR-3) and a subsequent decline leading to severe depletion at 72 h (−70% in SKOV3, −67% in OVCAR-3) (Figure 2). A similar down-regulation of DAG is also seen when cells are exposed to C75, the other FASN-inhibitor (Supplementary Figure 2). Our data corroborate recent findings [20] demonstrating that downregulation of DAG is essential for growth inhibition by FASN-inhibitors.

**Figure 3:**
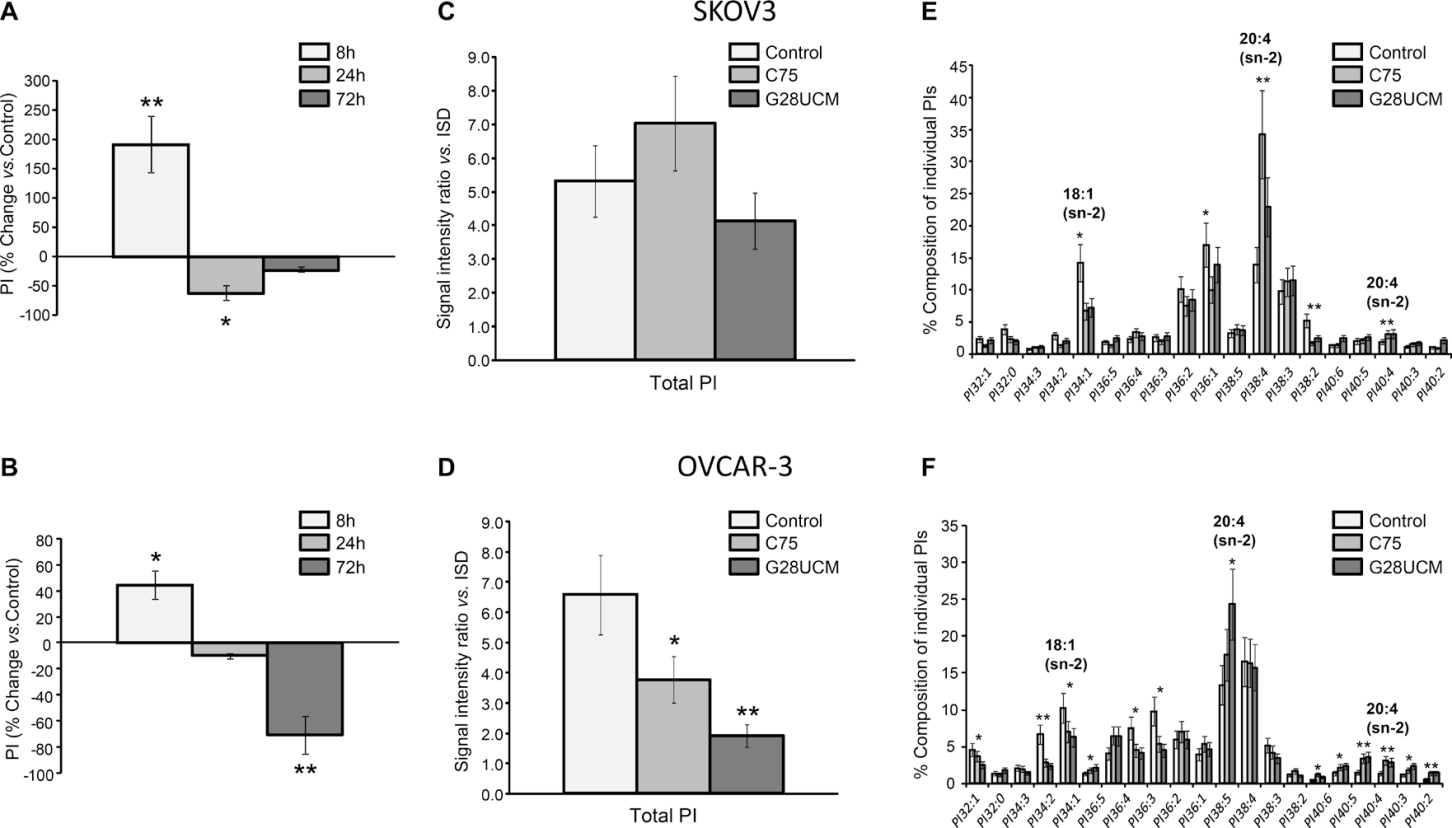
FASN-inhibitors G28UCM and C75 down-regulate total phosphatidylinositol (PI) levels and alter the composition of individual PI species in SKOV3 (upper row-A, C, E) and OVCAR-3 (lower row-B, D, F) cells as demonstrated by MALDI-QIT-TOF-MS/MS analysis (**A, B**) Time-course analyses revealed similar ‘bi-phasic response profiles’ of total PI in both cell lines with initial up-regulation at 8 h followed by sharp reductions at 24 and 72 h of exposure to G28UCM relative to untreated control cells (horizontal 0-line). (**C, D**) Long-term reduction of PI levels after 72 h treatment was less pronounced in SKOV3 (C) than in OVCAR-3 cells (D). ISD internal lipid standards. (**E, F**) Similar drug-induced changes in the composition of individual PI species were seen after 72 h in both SKOV3 (E) and OVCAR-3 (F) cells including reduction of MUFAs (e.g. OA, 18:1) and enrichment of PUFAs (e.g. arachidonic acid, 20:4). C75 and G28UCM were applied at concentrations that block growth by 60–70% after 72 h (40 μM for SKOV3 and 20 μM for OVCAR-3) [16]. For further details see also Supplementary Table 1. Means ± SD, n = 3. *p < 0.05, **p < 0.01 vs. untreated control cells, one-way ANOVA and Scheffe-test.

**Figure 4:**
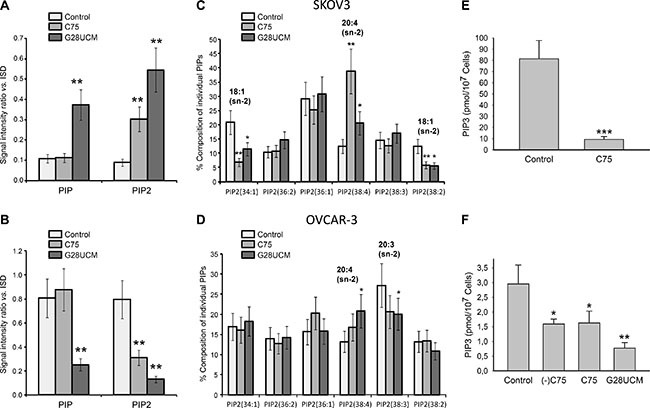
The effects of FASN-inhibitors G28UCM and C75 on the phosphorylated derivatives of phosphatidylinositol [PIP = PI(4)P, PIP2 = PI(4,5)P2, PIP3 = PI(3,4,5)P3] in SKOV3 (upper row A, C, E) and OVCAR-3 (lower row B, D, F) cells as demonstrated by MALDI-QIT-TOF-MS/MS analysis using ‘internal lipid standards’ (ISD) for quantitative evaluation (**A, B**) FASN-inhibition causes up-regulation of PIP and/or PIP2 in SKOV3 (A) but down-regulation in OVCAR-3 (B). (**C, D**) Yet, similar drug-induced changes in composition of PIP2 were seen in both cell lines including depletion of lipids containing 18 : 1 and enrichment of those containing 20 : 4 fatty acids. The most prominent fatty acid residues esterified to the sn-2 position of the glycerol backbone of the PIP2 molecules are indicated. Culture conditions were as in Figure 3. (**E, F**) Exposure for 24 h to FASN-inhibitors C75, (−)C75, or G28UCM down-regulates the amount of PIP3 in SKOV3 (E) and OVCAR-3 (F) cells relative to vehicle control (< 0.1% DMSO) as demonstrated by a competitive PIP3 mass ELISA. Means ± SD, n = 3. *p < 0.05, **p < 0.01, ***p < 0.001 vs. vehicle control cells, one-way ANOVA followed by Scheffe test (A–D and F) or Student‘s t test (E).

While oncogenic functions of FASN, DAG, PKC, PIPs, and PI3K-mTORC1 have already been demonstrated, the influence of FASN(-inhibitors) on phosphatidylinositol (PI) metabolism and signaling in cancer is still unknown [10, 21–23]. To address these issues we employed a matrix-assisted laser desorption/ionization (MALDI)-hybrid quadrupole iontrap (QIT)-time-of-flight (TOF)-tandem-mass spectrometry(MS/MS) based lipidomics screening approach [24]. A transient rise of total PI after 8 h treatment with G28UCM is followed by a sharp decline (Figure 3A, 3B). PI regulation is seen with both inhibitors in both cell lines, albeit it is less pronounced in SKOV3 (*cf*. Figure 3A, 3C with Figure 3B, 3D). MALDI-MS/MS fragment ion analysis was used to determine the fatty acid composition of the individual PI species. Approximately 20 peaks representing the most prominent PI species are found in the mass range between *m/z* 800–920 (Supplementary Table 1 and Supplementary Figure 3). Lipid species containing mainly OA (18:1(9Z)) are decreased, while those containing PUFAs like arachidonic acid (20 : 4 (5Z,8Z,11Z,14Z)) are increased after FASN-inhibition (Figure 3E, 3F). PI is precursor of all PI phosphates. Here we focus on PI 4-phosphate (PIP), PI 4,5-bisphosphate (PIP2), and PI 3,4,5-trisphosphate (PIP3). These phospholipids control crucial steps in cancer cell signaling [25]. FASN-inhibitors elevate PIP and/or PIP2 levels in SKOV3, but diminish them in OVCAR-3 cells (Figure 4A, 4B). Nevertheless, the drug-mediated changes of the FA composition of PIP2 are similar in both cell lines and correspond to those seen in PI, i.e. enrichment of PUFAs (e.g. arachidonic acid) and depletion of MUFAs (e.g. OA) (Figure 4C, 4D and Supplementary Figure 4). PI3K activates the PI3K-mTOR signaling cascade by phosphorylating PIP2 to PIP3. Using a PIP3-specific ELISA we demonstrate that FASN-inhibitory drugs strongly diminish the cellular amount of PIP3 in OC cells (Figure 4E, 4F).

### FASN-inhibitors impair GRB2 recruitment to the EGF receptor

**Figure 5:**
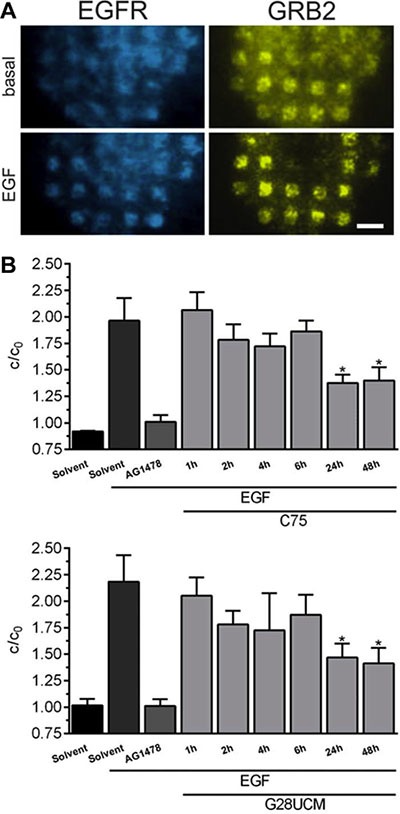
FASN-inhibitors C75 and G28UCM counteract EGF-induced recruitment of GRB2 to EGFR (**A**) Representative total internal reflection fluorescence microscopy images of a SKOV3 cell co-transfected with EGFR-CFP and GRB2-YFP and grown on an anti-EGFR antibody coated micro-biochip showing the specific recruitment of GRB2 to EGFR enriched regions after EGF stimulation (17 nM, 15 min; lower row) relative to basal interaction (upper row). Scale bar = 9 μm. (**B**) Quantitative evaluation of the YFP fluorescence contrast reveals that pretreatment with FASN-inhibitors (40 μM, 0–48 h) counteracts EGF-induced binding of GRB2 to EGFR in SKOV3 cells. c/c0 describes the ratio of YFP-fluorescence within (c) and between (c0) EGFR enriched regions. Error bars are based on the SE of > 15 analyzed cells from two individual experiments, respectively. *p < 0.05 vs. cells treated with EGF alone, one-way ANOVA and Scheffe-test.

Signal initiating molecules such as the epidermal growth factor (EGF) receptor (EGFR) tyrosine kinase are embedded in multi-molecular complexes that are anchored in membrane microdomains known as lipid rafts. FASN-blockade lowers the membrane lipid content. This leads to decomposition of the lipid rafts [26] and to reduced receptor phosphorylation [14]. Here we applied a live-cell micropatterning assay [27–29] to evaluate the role of FASN-blockade on recruitment of GRB2 to EGFR. SKOV3 cells co-expressing EGFR-CFP and GRB2-YFP were grown on anti-EGFR antibody coated micro-biochips and accumulation of GRB2 in EGFR-microdomains was observed by total internal reflection fluorescence microscopy. Figure 5 reveals that EGF (17 nM, 15 min)-induced tethering of GRB2-YFP to EGFR-CFP is prevented not only by pretreatment with the EGFR-inhibitor AG1478 (10 μM, 4 h), but also by the FASN-inhibitors C75 and G28UCM. Decrease of the fluorescence contrast (c/c_0_) is already observed after 2 hours and reaches statistical significance after 24 hours of treatment (Figure 5B).

### HIF-1α/REDD1 and AMPK link FASN-blockade to mTORC1 suppression and growth inhibition

**Figure 6:**
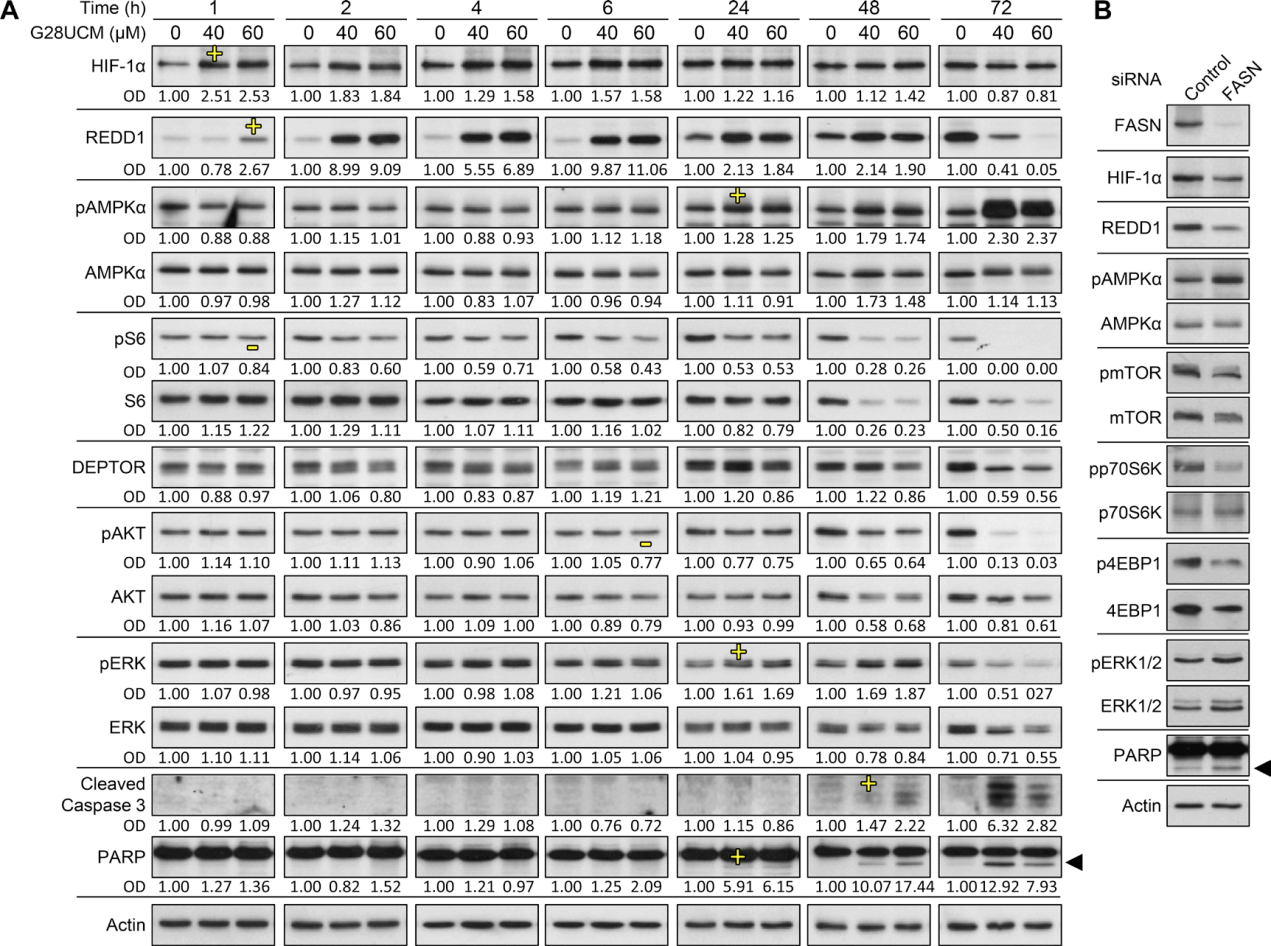
Pharmacologic inhibition or genetic knock-down of FASN impedes PI3K-mTORC1 signaling and induces apoptosis in OC cells as demonstrated by Western blot analysis (**A**) G28UCM-treated SKOV3 cells reveal early upregulation of HIF-1α and REDD1 and concurrent inhibition (de-phosphorylation) of the mTORC1 downstream effector ribosomal S6 protein (pS6). Subsequently, REDD1 and pAKT become decreased, whereas pAMPKα gets increased. Levels of pERK peak at 24–48 h. Cleavage products of caspase 3 and PARP1 indicating apoptosis occur after 24 h of treatment. ‘-‘ First indication of down-regulation, ‘+’ first indication of up-regulation. OD optical density of specific band relative to actin band normalized to untreated control cells (0 μM G28UCM). (**B**) FASN siRNAs abrogate FASN protein expression in A2780 cells 72 h post-transfection and down-regulate HIF-1α, REDD1, pmTOR, mTOR, pp70S6K, p4EBP1, and 4EBP1, whereas levels of pAMPKα, pERK1/2, ERK1/2, and cleaved PARP increase. Actin was used as loading control. ◄cleaved PARP.

**Figure 7:**
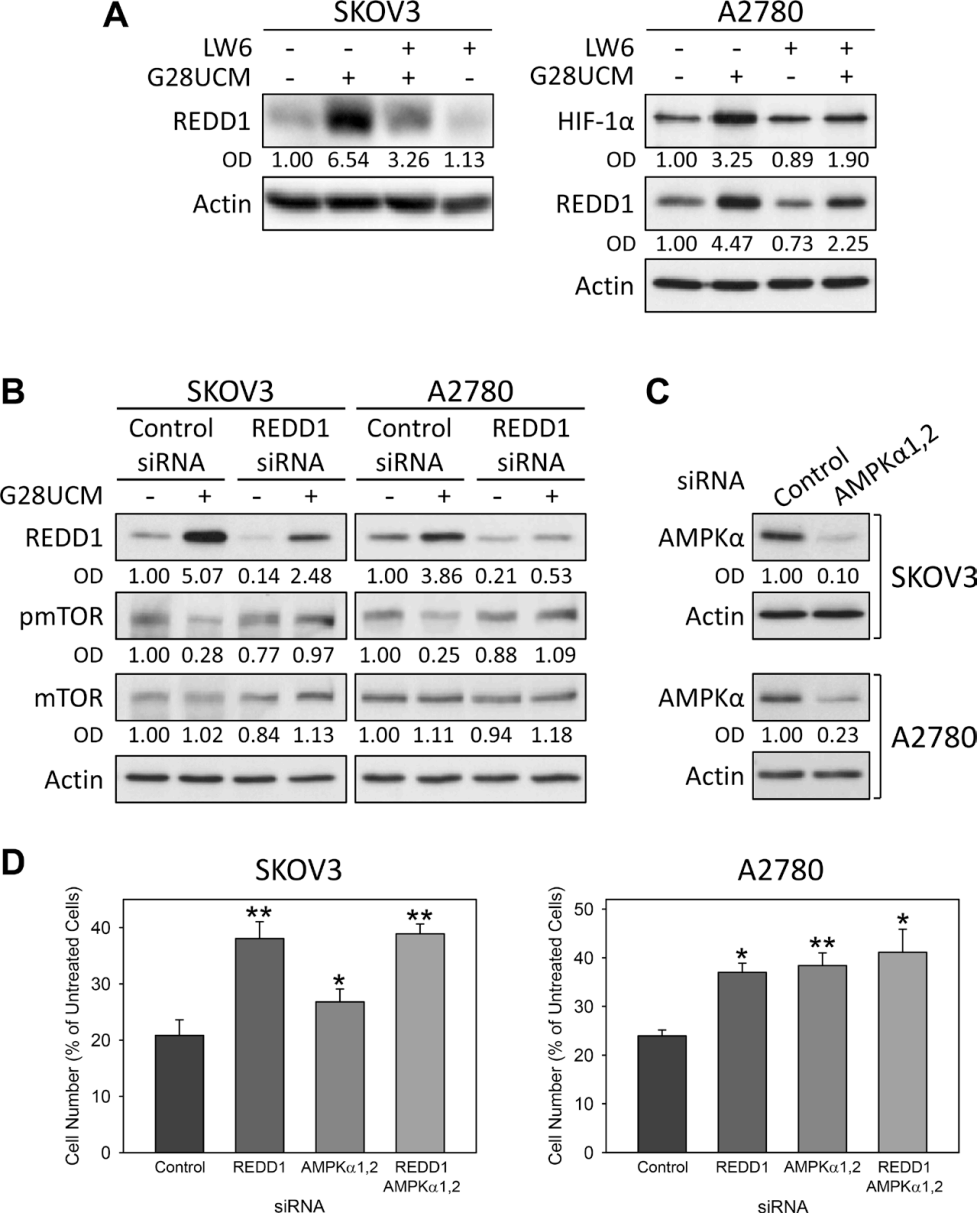
HIF-1α-mediated up-regulation of REDD1 and phosphorylation of AMPKalpha(greek symbol) are crucial for the anticancer effect of FASN-inhibitors in OC cells as demonstrated by Western blot analysis (A–C) and formazan dye assay (D) (**A**) The HIF-1α inhibitor LW6 prevents G28UCM-mediated up-regulation of HIF-1α and REDD1. SKOV3 (left) or A2780 (right) cells received 2 or 10 μM LW6 for 18 h followed by a 6 h-exposure to 40 or 20 μM G28UCM, respectively. (**B, C**) REDD1-(B), AMPKα1- and AMPKα2-specific siRNAs (C) dampen the expression of their respective target genes and (B) prevent G28UCM-mediated (40 μM, 4 h) down-regulation of pmTOR in SKOV3 and A2780 cells. OD, optical density of specific band relative to actin band normalized to untreated control cells. (**D**) siRNA knock-down of REDD1 or AMPKα1,2 alone or together is lowering the growth inhibitory effect of G28UCM (40 μM, 96 h) in SKOV3 and A2780 cells. Means ± SD, n = 3. *p < 0.05, **p < 0.01 target siRNA vs. control siRNA, one-way ANOVA followed by Scheffe test.

Western blotting was used to examine FASN-blocker-mediated inhibition of PI3K-AKT-mTORC1. Thereby, we aimed to identify effector molecules that directly link FASN pathways to mTORC1. In SKOV3 cells treated with G28UCM we observed a rapid dose-dependent induction of HIF-1α and of its target gene, the stress response protein REDD1 (DDIT4, RTP801) (Figure 6A). Figure 7A reveals that blockade of HIF-1α with the specific inhibitor LW6 prevents G28UCM-mediated induction of REDD1 indicating that REDD1 up-regulation is mediated by HIF-1α. Accordingly, both proteins accumulate within the first hour of FASN-inhibition. However, after 72 h, both HIF-1α and REDD1 are decreased relative to control cells (Figure 6A). Concurrently, phosphorylation of AMPK, which signifies shortage of bioenergy, becomes stimulated. Both REDD1 and pAMPK are repressors of mTORC1. Consequently, phosphorylation of the mTORC1 effector ribosomal protein S6 is decreasing as soon as REDD1 is up-regulated and remains low due to ensuing suppression through pAMPK. Reduction of mTOR and of the mTOR binding partner DEPTOR has been observed after 2–3 days of treatment confirming robust impairment of the mTOR pathway (Figure 6A, 6B). Cleaved fragments of caspase 3 and PARP–two hallmarks of apoptosis–occur approximately 24 h post-treatment. At that time mTORC1 activity has already been impaired (Figure 6A). Similar molecular changes are found in G28UCM-and C75-treated A2780 or OVCAR-3 cells (Supplementary Figure 5). Moreover, the effects are independent of the presence or absence of exogenous growth factors and can also be observed after RNAi knock-down of the FASN gene (Figure 6B and Supplementary Figure 6). Genetic knock-down of REDD1 or AMPKα1/2 reverts G28UCM-mediated mTOR dephosphorylation and growth arrest (Figures 7B–7D). However, concurrent knock-down of both REDD1 and AMPKα1/2 does not yield stronger reversal of growth inhibition (Figure 7D). These data indicate that REDD1 and AMPK are interfering with the very same pathway, namely mTORC1, which essentially contributes to cell death. In contrast, carboplatin, a DNA-cross linking chemotherapeutic drug with an unrelated mechanism of action, induces growth arrest and apoptosis, but does not elevate HIF-1α, REDD1 or pAMPKα, nor does it impede phosphorylation of S6 downstream of mTORC1 (Supplementary Figure 7).

### Combining FASN-inhibitors with a MEK-inhibitor augments growth inhibition in OC cells

**Figure 8:**
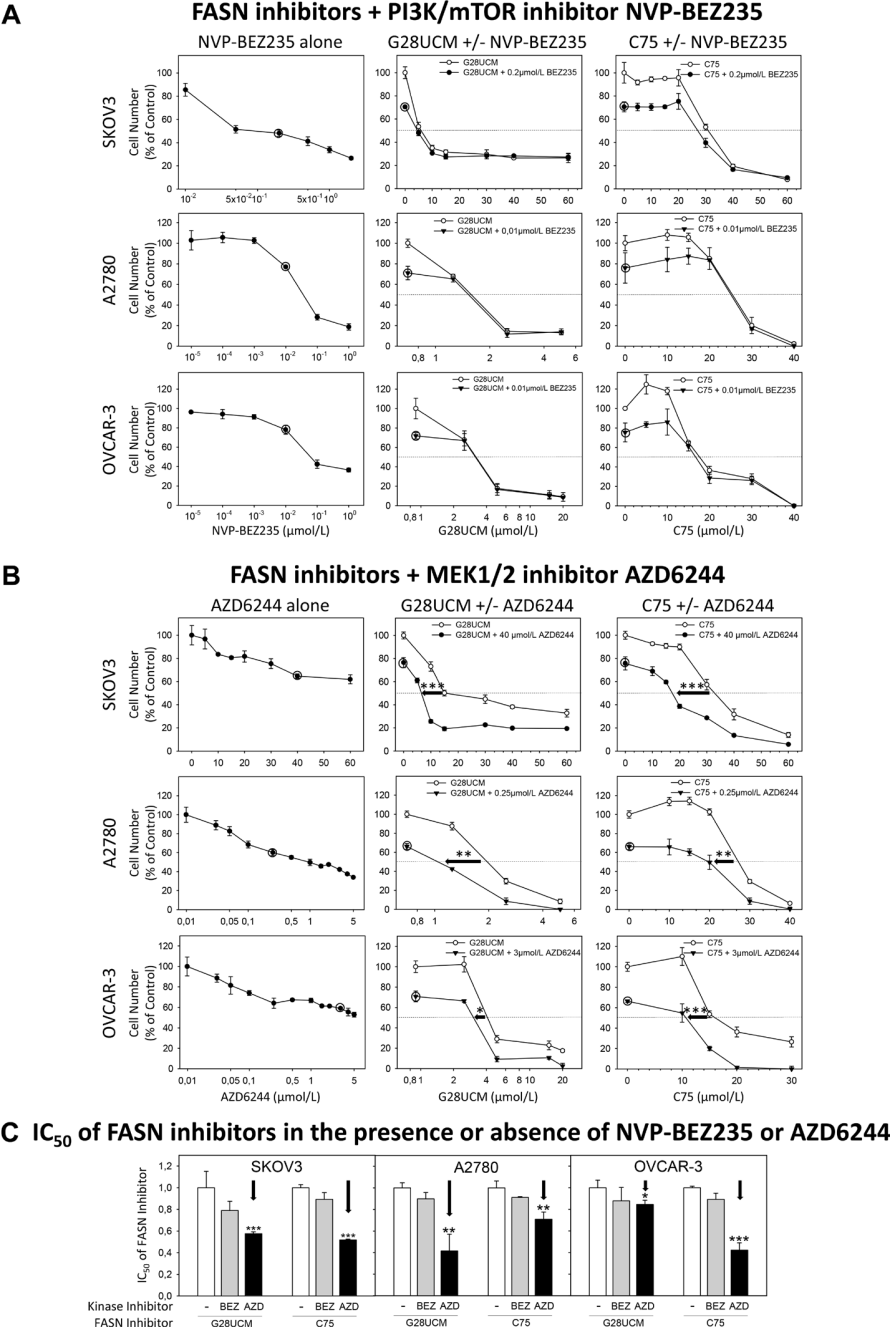
The effect of the dual PI3K/mTOR kinase inhibitor dactolisib/NVP-BEZ235 and of the MEK1/2 kinase inhibitor selumetinib/AZD6244 on the anti-proliferative effects of the FASN-blockers G28UCM and C75 as demonstrated by formazan dye assay SKOV3, A2780 or OVCAR-3 cells were treated for 72 h with NVP-BEZ235 (**A**) or AZD6244 (**B**) alone (left charts), or with G28UCM (center charts) or C75 (right charts) alone or together with NVP-BEZ235 or AZD6244. A circle symbol as given in the original manuscript and in the corresponding diagrams marks the fixed concentration of the kinase inhibitors chosen for combination with various doses of the FASN-inhibitors. In (**C**) a comparison of the relative IC50 values of the FASN-blockers G28UCM and C75 in the absence (arbitrarily set at y = 1) or presence of NVP-BEZ235 (BEZ) or AZD6244 (AZD) is given. Note: only AZD6244 can significantly lower the IC50 values of the FASN-inhibitors G28UCM and C75 (←). Means ± SD, n = 3. *p < 0.05, **p < 0.01, ***p < 0.001 presence (AZD) vs. absence of AZD6244 (−), one-way ANOVA followed by Scheffe test.

Inactivation of PI3K-mTORC1 is accompanied by compensatory stimulation of MAPK as evidenced by elevated levels of pERK after 1 and 2 days of treatment (Figure 6A, Supplementary Figure 5D). This FASN-inhibitor-dependent loosening of PI3K-MAPK cross-repression [30] prompted us to determine the effect of FASN-blockers combined with kinase inhibitors on OC cell growth. A large proportion of OC reveals hyper-activation of the PI3K-mTORC1 pathway [6]. Consequently, these cells are highly sensitive to dual PI3K-mTOR-inhibitors such as dactolisib/NVP-BEZ235, but not to MAPK pathway-inhibitors like MEK1/2-blocker selumetinib/AZD6244 (Figure 8A, 8B). Co-exposure to moderately effective doses of NVP-BEZ235 (0.2 μM in SKOV3 and 0.01 μM in A2780 and OVCAR-3) cannot improve the anticancer effect of the FASN-blockers (Figure 8A), whereas combination with AZD6244 (40 μM in SKOV3, 0.25 μM in A2780 and 3 μM in OVCAR-3) significantly increases the FASN-inhibitor sensitivity (Figure 8B). Accordingly, the IC_50_ values of G28UCM and C75 are significantly reduced in the presence of AZD6244, but not of NVP-BEZ235 (Figure 8C). This indicates that the mechanism of action of FASN-blockers is mediated through PI3K-mTORC1 inactivation. However, the anti-proliferative efficacy of FASN-inhibitors is impaired by compensatory cross-induction of RAF-MEK-ERK.

## DISCUSSION

Inhibition of FASN leads to FA depletion and accumulation of malonyl-CoA. The relative contribution of both events to FASN-inhibitor toxicity has been discussed [22, 31] and obviously depends on the type of inhibitor (Figure 1A–1C). Inhibitor-induced shortage of required reaction product can be uncoupled from inhibitor-induced buildup of toxic levels of substrate by blocking the upstream lipogenic enzyme acetyl-CoA carboxylase. This enzyme provides FASN with malonyl-CoA. Therefore, Acetyl-CoA carboxylase-inhibitors such as TOFA cause FA depletion in the absence of high levels of malonyl-CoA [31]. Interestingly, TOFA reveals only low/moderate cytotoxicity in ovarian cancer cells (Figure 1D) suggesting that ,besides FA depletion, malonyl-CoA elevation essentially contributes to the cytotoxicity of FASN-inhibitors. Nevertheless, supplementation of OA has been found to significantly alleviate growth inhibition by FASN-blockers and partially restores cancer cell survival, whereas supply of the primary enzyme product PA is rather toxic (Figure 1B, 1C). This indicates that availability of OA is crucial for sustained OC cell growth and that OC cells are not able to process exogenous PA properly. In addition, we observed that FASN-blockers alter the level of DAG and PI in an opposing biphasic manner. Early depletion of DAG concurs with a rise in PI followed by accumulation of DAG and reduction of PI. Eventually, the cells turn into a state of severe deficiency of both DAG and PI (*cf*. Figures 2 with 3A, 3B). This suggests that the drug-induced shortage of membrane phospholipids is initially balanced by intense conversion of DAG into PI. Eventually, however, the cells cannot prevent the lipid content to fall below a crucial threshold (see also Supplementary Figure S4). This stage is characterized by an ‘unsaturated’ membrane lipid phenotype, which is reflected by low MUFA-/high PUFA-content in PI and its phosphorylated derivatives. Previous studies have shown that cancer cell membranes in general tend to have a higher degree of saturation, thus rendering them more resistant to oxidative stress [32]. Hence, our study demonstrates that FASN-inhibition restores a membrane lipid phenotype that is reminiscent of non-malignant cells characterized by a low amount of saturated lipids.

FASN lies at the crossroads between catabolic and anabolic pathways. It controls homeostasis, energy balance and cell growth, and interacts with oncogenic signaling [22]. We recently observed that FASN-inhibition impairs ERBB-PI3K-mTORC1 signaling in OC [14, 15]. Yet, the underlying mechanism(s) remain elusive. Here we describe a number of events that lead to prompt inactivation of ERBB-PI3K-mTORC1 upon blockade of FA synthesis. In particular, FASN-inhibition causes down-regulation of membrane phospholipids. It reduces the level of EGFR (ERBB)-receptor expression [14] and inhibits binding of GRB2 to EGFR (Figure 5B). This results in a decrease of signal activity upstream of PI3K. PIP2 is known to give rise to two signaling lipids: 1. DAG, which stimulates RAF and PI3K and 2. PIP3 generated by PI3K. These two lipids are located upstream and downstream of PI3K, respectively. We observed that FASN-inhibitory drugs down-regulate DAG corroborating previous data [20]. Moreover, we show for the first time that FASN-inhibitors lead to a significant deficit in PIP3 leading to inactivation of AKT-mTORC1, which provides yet another cogent explanation for the observed FASN-inhibitor-dependent PI3K pathway suppression. Accordingly, mTORC1 downstream target ribosomal protein S6 becomes dephosphorylated. In addition, we observed that FASN-blockers activate cell stress proteins such as HIF-1α and REDD1, which are known to repress mTORC1. A similar REDD1 involvement has recently been observed when cells are exposed to the anti-lipogenic compound orlistat [32]. In our setup, up-regulation of REDD1 is transient and is followed by elevated pAMPK, another negative regulator of mTORC1, which gets activated when bioenergy is short. Quiescence of PI3K-mTORC1 was found to precede FASN-inhibitor-induced cleavage of caspase 3 and PARP, two markers of apoptosis. Accordingly, knockdown of either REDD1 or AMPK renders phosphorylation/activity of mTOR (pmTOR) resistant to FASN-blockers and relieves drug-induced growth inhibition (Figure 7B–7D). Attenuation of ERBB-PI3K-mTORC1 activity upon FASN blockade has been seen in all OC cell lines (SKOV3, A2780, OVCAR-3) independent of the mode of FASN inhibition (G28UCM, C75, FASN siRNAs) and independent of the presence or absence of ERBB-stimulating growth factors such as EGF and heregulin (HRG)-β1 (Figure 6 and Supplementary Figures 5 and 6). In contrast, the standard cytotoxic drug carboplatin induces apoptosis without elevating HIF-1α, REDD1 or pAMPKα, and it does not diminish phosphorylation of S6 protein. This indicates that down-regulation of mTORC1-S6 activity is specifically required for apoptosis induction by FASN-inhibitors but not by other drugs such as carboplatin. Combination of G28UCM or C75 with the dual PI3K/mTOR inhibitor NVP-BEZ235 (Figure 8) or with statins (not shown), which inactivate mTORC1 as well [33], fails to strengthen the G28UCM- or C75-mediated anticancer response in SKOV3, A2780 or OVCAR-3 cells. This indicates FASN-inhibitor-mediated mTORC1 silencing is already maximal and additional blockade with yet another PI3K/mTOR inhibitor is not productive anymore. Recent evidence suggests that mTORC1-S6 activity may convert a reversible cell cycle arrest into an irreversible one, which typically occurs during cell senescence (geroconversion). Thus, in addition to its usual function as oncogenic driver, mTORC1-S6 may also act as inducer of cell aging when the cycle machinery has been halted by other means [34–36]. Phosphorylation of the MAPK ERK is increased when pAKT levels drop. Most likely, this is due to release of PI3K-AKT cross-inhibition of RAF-MEK-ERK. Accordingly, concurrent blockade of the MEK-ERK axis by MEK1/2 inhibitors such as AZD6244 or by upstream ErbB inhibitors [14] increase the sensitivity against FASN-blockers. Thus FASN-blocker-mediated decrease of PI3K-mTORC1 signaling results in untightening of cross-inhibition of MAPK thereby enabling compensatory MAPK activation, which lowers the anticancer effect of FASN-blocking agents. Further clinical development and evaluation of combinations of FASN-targeting drugs with MAPK pathway inhibitors are therefore warranted.

## MATERIALS AND METHODS

### Cell culture and drugs

OC cell lines HOC-7 (R.N. Buick, Toronto, Canada), OVCAR-3, SKOV3 (ATCC, Manassas, VA) and A2780 (M. Krainer, Medical University Vienna, Austria) were maintained in α-MEM or RPMI1640 [37]. Media were supplemented with 10% fetal calf serum (FCS), 100 IU (μg)/ml penicillin-streptomycin and 2 mM glutamine (Gibco, Karlsruhe, Germany). Cells were maintained at 37°C, 5% CO_2_ and 95% humidity and were tested for absence of viral/bacterial/fungal/mycoplasmal infection (Venor GeM, Minerva Biolabs, Berlin, Germany). The species origins were proven by species-PCR, and cell line identities were examined by fluorescent nonaplex-PCR of short tandem repeat markers in summer of 2014 (DSMZ, Braunschweig, Germany). PA (16:0) and OA (18:1(9Z))(Sigma, St. Louis, MO) dissolved in ethanol were complexed with bovine serum albumin in Dulbecco's phosphate buffered saline [38] and diluted in growth media to final concentration of 70 μM. Recombinant human EGF and heregulin-β1 (HRG) were purchased from Sigma and Thermo Fisher Scientific (Fremont, CA), respectively. EGFR inhibitor AG1478 (Calbiochem, San Diego, CA), PI3K/mTOR dual blocker dactolisib/NVP-BEZ235, MEK1/2 inhibitor selumetinib/AZD6244 (ChemieTek, Indianapolis, IN), HIF-1α inhibitor LW6 (Calbiochem), FASN-inhibitors C75 (racemic mixture of (−)- and (+)-enantiomers, Sigma), (−)-C75 (F.G. Hegardt, D. Serra, Barcelona, Spain) [39], G28UCM (R. Colomer, M.L. López Rodríguez, Madrid, Spain) [40, 41] and TOFA (Calbiochem) were dissolved in DMSO, whereas carboplatin (Selleckchem, Houston, TX) was dissolved in sterile water. Stocks were diluted 1:500 or 1:1 000 in media.

### Malonyl-CoA assay

SKOV3 cells (2 × 10^4^/cm^2^) exposed for 0–72 h to 40 μM C75 or G28UCM were lysed in ice-cold lysis buffer (20 mM MOPS, pH 7.0; 2 mM EGTA; 5 mM EDTA; 1% Triton X-100), sonicated (4 strokes at median amplitude, 10 sec each, 4°C) and centrifuged (1 500 × g, 15 min, 4°C). The supernatants containing 1.3 mg protein were subjected to the Malonyl CoA BioAssay ELISA following manufacturer's instructions (United States Biological, Salem, MA).

### Cell proliferation

Cells (500–3 000/well, 96-well plate) attached overnight before media containing 5% FCS ± drugs were added. Cell numbers were determined using a formazan dye assay (Biomedica, Vienna, Austria) as described [42].

### Apoptosis assay

SKOV3 cells (2 × 10^4^/cm^2^) attached overnight before media containing 5% FCS ± drugs were added. At the end of treatment, cells were trypsinized and counted. Ten thousand cells per sample were lysed in proprietary lysis buffer and further processed for detection of apoptosis using the Cell Death Detection ELISA^plus^ system from Roche Diagnostics GmbH (Vienna, Austria) following the manufacturer's protocol.

### Lipid extraction and thin-layer chromatography

A modified two-step organic solvent extraction protocol [43] was used to enrich PIs and PIPs. Briefly, cold CHCl_3_ :MeOH (50:50 [v/v]) added to the washed cell pellet, vortexed and centrifuged (2 000 × g, 5 min) yielded a supernatant designated ‘Extract 1’. The cell pellet was extracted again with CHCl_3_ : MeOH (70:30 [v/v], containing 0.3% aqueous HCl), sonicated (15–30 sec) and vortexed (5 min). 1N HCl was added, vortexed (1–2 min) and centrifuged (12 000 × g, 5 min). The lower organic phase represents ‘Extract 2’. This procedure leads to efficient separation of > 85% PI in ‘Extract 1’ and > 95% PIP and PIP2 in ‘Extract 2’ (Supplementary Figure 8). For thin-layer chromatography, aliquots of both extracts were pooled and the lipids were separated as described [16].

### Mass spectrometry (MS)

Mass spectra were recorded using an AXIMA-CFRplus (Shimadzu, Manchester, UK) curved-field reflectron TOF mass spectrometer equipped with a 337-nm pulsed nitrogen laser in the negative mode using 9-aminoacridine as matrix [44]. Ion acceleration voltage was 20 kV and the reflectron analyzer was at 25 kV. For structural identification of lipid molecules a hybrid quadrupole iontrap (QIT)-TOF tandem-mass spectrometer (MS/MS; AXIMA-Resonance, Shimadzu) was used. Acquisition was in the low-mass range (*m*/*z* 300–1 000) and high-resolution (R = 1 000) ion selection modes for MS/MS experiments of monoisotopically selected precursor ions using low-energy collision induced dissociation with argon as the collision gas. Each spectrum shown represents the accumulation of 300–500 single laser shots. An external calibration based on the exact mass values of the [M-H]^-^ ions of defined lipid standards was applied. The structural identity of the different lipid species detected in the samples was confirmed by MALDI-MS/MS spectra of authentic reference compounds (Supplementary Figure 9). Data processing was performed by Launchpad 2.9.3 software (Shimadzu) using the Savitzky-Golay smoothing algorithm.

### PIP3 mass ELISA

PIP3, unlike PIP and PIP2, was not amenable to MS analysis–probably due to the high negative charge of PIP3 causing co-segregation with the protein fraction. This lipid had therefore to be analyzed with a different technology. SKOV3 and OVCAR-3 cells plated at 2.5 × 10^6^ and 5 × 10^6^, respectively, in 150 mm dishes were grown in α-MEM containing 5% FCS and treated for 24 h with the indicated concentrations of the FASN-inhibitors. For detection of PI3K activity the cellular amount of its reaction product PIP3 was directly quantitated using a competitive PIP3 mass ELISA system following the manufacturer's protocol (Echelon, Salt Lake City, UT). Thereby PIP3 extracted from 10^7^ cells is first incubated with a PIP3-detector protein. The PIP3/PIP3-detector protein-complex is then added to a PIP3-coated microplate for competitive binding. A peroxidase-linked secondary reagent and a colorimetric substrate are used to visualize the PIP3-detector protein bound to the plate. The colorimetric signal is inversely proportional to the amount of PIP3 extracted from the cells.

### Micropatterning assay

For detection and quantitation of the effects of FASN-inhibition on GRB2 recruitment to the membrane-bound EGFR signaling complex a protocol applying micropatterning followed by total internal reflection fluorescence microscopy and image analysis was executed as described [19, 27–29].

### Western blotting

Cells plated in media containing 5% FCS were treated with solvent (0.1% DMSO) in the absence or presence of various concentrations of FASN-inhibitor for 1 to 72 h. After lysis, proteins were subjected to SDS–PAGE, blotted and immunostained as described [45, 46] using anti-FASN (BD Biosciences, San Jose, CA; 1:500), anti-AKT, anti-pAKT, anti-AMPKα, anti-pAMPKα, anti-DEPTOR, anti-ERK, anti-pERK, anti-HIF-1α, anti-mTOR, anti-pmTOR, anti-p70S6K, anti-pp70S6K, anti-S6, anti-pS6, anti-4EBP1, anti-p4EBP1, anti-cleaved caspase 3, anti-PARP1 (Cell Signaling Technology, Boston, MA; 1 : 500–1:3 000), anti-REDD1 (Proteintech, Manchester, UK; 1:1 000), and anti-actin (Santa Cruz Biotechnology, Dallas, TX; 1:1 000). Secondary antibodies were peroxidase-labelled donkey-anti-rabbit (Promega, Madison, WI) or donkey-anti-goat IgG (Santa Cruz Biotechnology) at 1:15 000, or chicken-anti-mouse IgG (Santa Cruz Biotechnology) at 1:10 000. Detection was by enhanced chemiluminescence.

### RNA interference

A2780 or SKOV3 cells were transiently transfected with 75 nM of FASN-, REDD1- and/or AMPKα1- and AMPKα2-siRNAs according to the manufacturer's instructions (Dharmacon, Lafayette, CO). Briefly, 3 × 10^5^ cells/35-mm dish were incubated in DharmaFECT-3 siRNA lipid transfection reagent either containing corresponding concentrations of siGENOME non-targeting siRNA #2 (D-001210-02) or containing a set of 4 siRNA species, each at a concentration of 18.75 nM, that target the mRNA of FASN or of the mTORC1 upstream repressors REDD1, AMPKα1, or AMPKα2, respectively (ON-TARGETplus SMARTpool FASN, L-003954-00; ON-TARGETplus SMARTpool REDD1, L-010855-01; ON-TARGETplus SMARTpool AMPKα1, L-005027-00; ON-TARGETplus SMARTpool AMPKα2,L-005361-00). Cells were cultured for 72 h at 37°C, 5% CO_2_, and 95% humidity in RPMI-1640 or α-MEM containing 5% FCS and 2 mM glutamine. FASN siRNA-transfectants were directly lysed, whereas the other transfectants were cultured and exposed for 96 h to 0.1% DMSO (vehicle) or 40 μM G28UCM and then subjected to a formazan dye cell proliferation assay (see above). Moreover, short-term (4 h) vehicle- or 40 μM G28UCM-treated transfectants were lysed and processed for SDS-PAGE and Western blotting to demonstrate efficient knockdown of baseline and FASN-inhibitor-induced target protein expression and its stabilizing effect on mTOR phosphorylation (see above). Data were obtained from unselected polyclonal transfectant populations.

## SUPPLEMENTARY MATERIALS FIGURES AND TABLES




