# Supplementary material for: Multi-level suppression of receptor-PI3K-mTORC1 by fatty acid synthase inhibitors is crucial for their efficacy against ovarian cancer cells
Source: Oncotarget. 2017 Jan 10;8(7):11600–13. doi: 10.18632/oncotarget.14591 (PMC5355289; doi:10.18632/oncotarget.14591)
Supplement: Supplementary file 1 [file oncotarget-08-11600-s001.pdf]

Multi-level suppression of receptor-PI3K-mTORC1 by fatty acid synthase inhibitors is crucial for their efficacy against ovarian cancer cells

Supplementary Materials

SUPPLEMENTARY REFERENCES

1. Veigel D, Wagner R, Stübiger G, Wuczkowski M, Filipits M, Horvat R, Benhamú B, López-Rodríguez ML, Leisser A, Valent P, Grusch M, Hegardt FG, García J, et al. Fatty acid synthase is a metabolic marker of cell proliferation rather than malignancy in ovarian cancer and its precursor cells. Int J Cancer. 2015; 136:2078–2090.
2. Kim Y, Shanta SR, Zhou LH, Kim KP. Mass spectrometry based cellular phosphoinositides profiling and phospholipid analysis: a brief review. Exp Mol Med. 2010; 42:1–11.

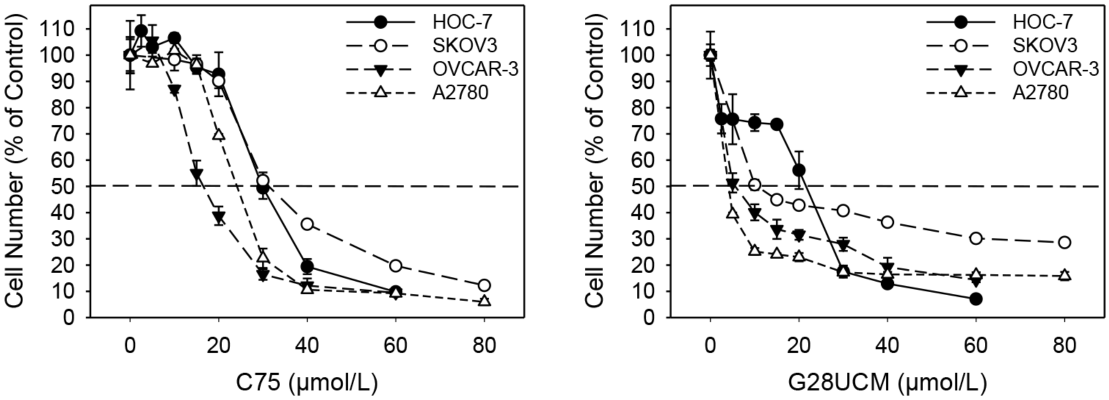

Supplementary Figure 1: The FASN-inhibitors C75 (left) and G28UCM (right) inhibit the *in vitro* growth of A2780, HOC-7, OVCAR-3 and SKOV3 ovarian cancer cells with different efficacy as demonstrated by formazan dye assay after 72 h of treatment. Fifty % growth inhibition is marked by the dashed horizontal line. Means ± SD, *n* = 3.

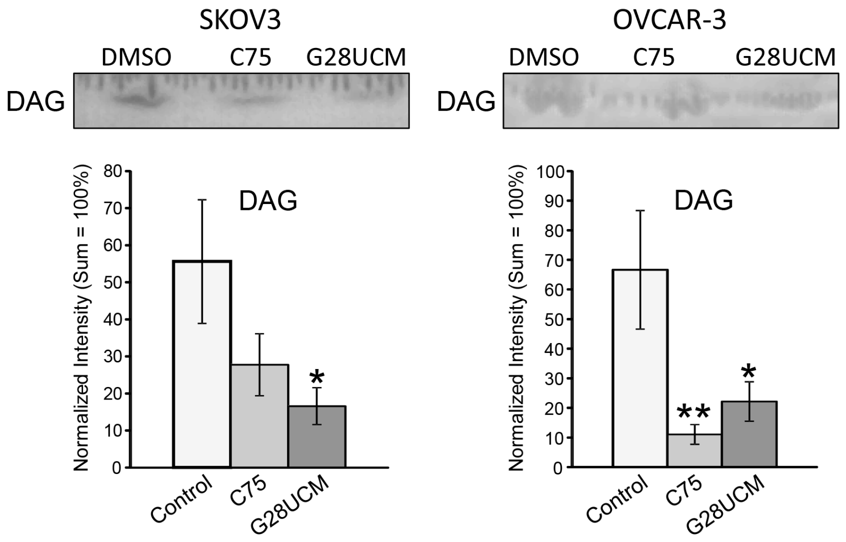

Supplementary Figure 2: Down-regulation of DAG is a general response to FASN inhibition as demonstrated by thin-layer chromatography in SKOV3 (left) and OVCAR-3 cells (right). Cells were exposed for 72 h to concentrations of C75 or G28UCM that block growth by 60–70% (40 µM for SKOV3 and 20 µM for OVCAR-3) [1]. Means ± SD, $n = 3$. $^*p < 0.05$, $^{**}p < 0.01$ *vs*. vehicle treated control cells, one-way ANOVA and Scheffe-test.

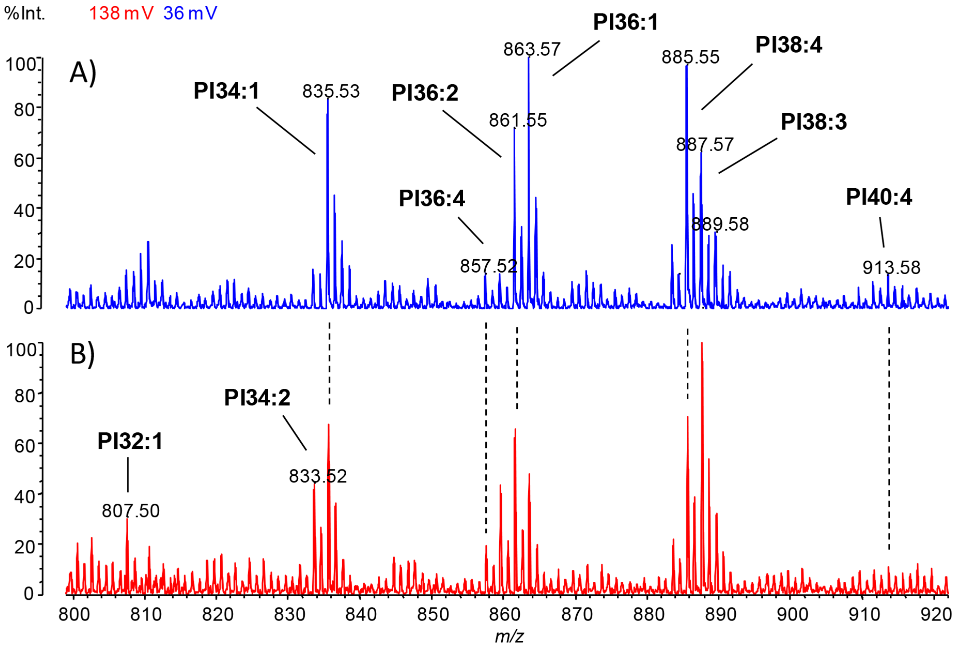

Supplementary Figure 3: MALDI-MS spectra of individual PI species detected within the m/z range of 800–920 in lipid extracts of (A) untreated SKOV3 and (B) untreated OVCAR-3 cells. Indicated are the most abundant lipid species based on identification by MALDI-QIT-TOF-MS/MS (see also Supplementary Table 1).

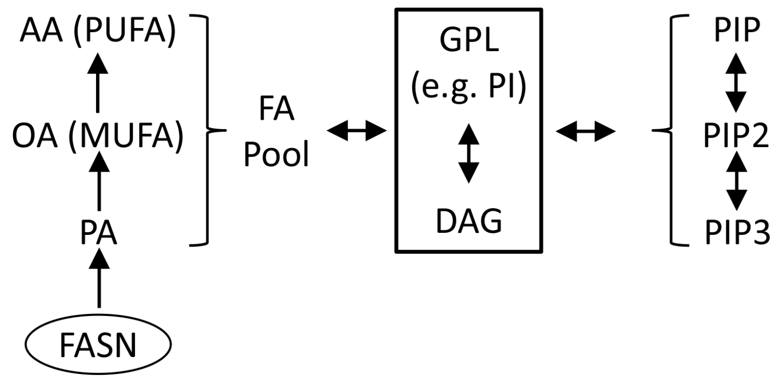

Supplementary Figure 4: Interplay between the major lipid classes investigated in this study. Remodeling between diacylglycerol and glycerophospholipids (e.g. phosphatidylinositol) plays a crucial role during FASN inhibition. Abbreviations: AA arachidonic acid, PUFA polyunsaturated fatty acids, OA oleic acid, MUFA monounsaturated fatty acids, PA palmitic acid, FASN fatty acid synthase, FA fatty acid, GPL glycerophospholipids, PI phosphatidylinositol, DAG diacylglycerol, PIP phosphatidylinositol 4-phosphate, PIP2 phosphatidylinositol 4,5-bisphosphate, PIP3 phosphatidylinositol 3,4,5-trisphosphate.

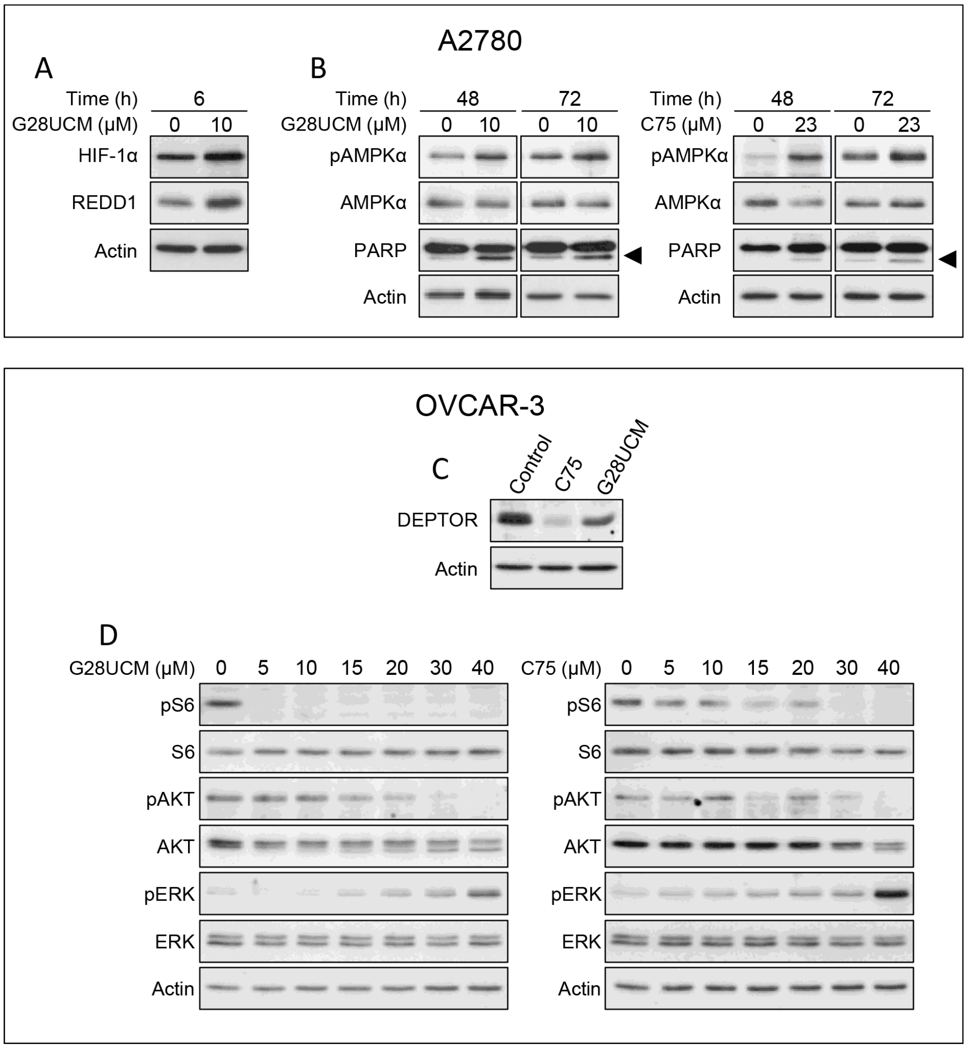

Supplementary Figure 5: FASN inhibitors G28UCM and C75 impede PI3K-mTORC1, but stimulate MAPK ERK signaling in A2780 (A, B) or OVCAR-3 (C, D) cells as demonstrated by Western blot analysis. (**A**, **B**) FASN inhibition induces the mTORC1 repressors HIF-1α, REDD1 and AMPK. (A) Early (6 h) up-regulation of HIF-1α and REDD1 and (B) delayed (48 h, 72 h) phosphorylation of AMPKα accompanied by cleavage of PARP. (**C**) Downregulation of the mTOR binding partner DEPTOR after 48 h exposure to 20 µM C75 or G28UCM. (**D**) Dose-dependent reduction of pS6 and pAKT and elevation of pERK after 48 h exposure to G28UCM or C75. Actin was used as loading control.

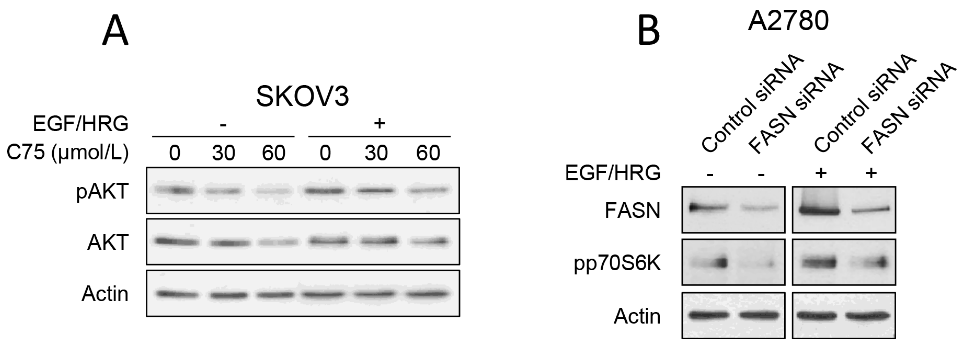

Supplementary Figure 6: Inhibition of FASN causes inactivation of the PI3K-mTORC1 pathway independent of the presence or absence of activating growth factors (100ng/ml EGF + 1nM HRG-β1, 20 min) as demonstrated by Western blot analysis. (**A**) SKOV3 cells were treated for 48 h with 0–60 µM C75, exposed to solvent (−) or growth factors (+) and then blotted against anti-pAKT or anti-AKT antibodies. (**B**) A2780 cells were transfected for 72 h with non-targeting control siRNAs or FASN siRNAs, challenged with solvent (−) or growth factors (+) and blotted against anti-pp70S6K antibody. Actin was used as loading control.

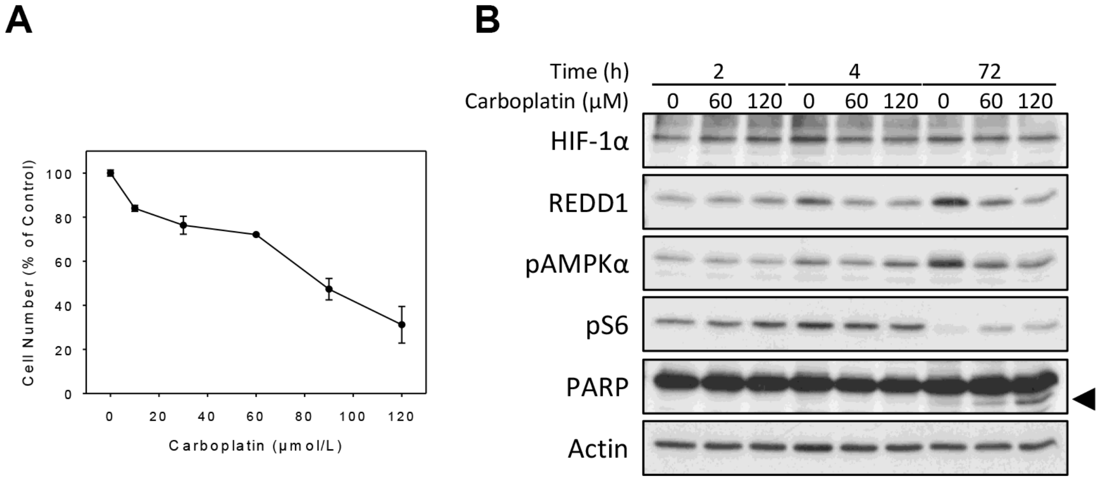

Supplementary Figure 7: Silencing of PI3K-mTORC1 signaling is not involved in the cytotoxic action of the standard chemotherapeutic drug carboplatin in SKOV3 cells. This compound inhibits *in vitro* growth and stimulates apoptosis, but does not elevate HIF-1α-, REDD1- or pAMPKα expression, nor does it downregulate pS6. (**A**) Formazan dye assay for evaluation of cell growth after 72 h drug exposure. (**B**) Western blot analysis of protein expression after 2, 4 or 72 h of treatment. Actin was used as loading control.

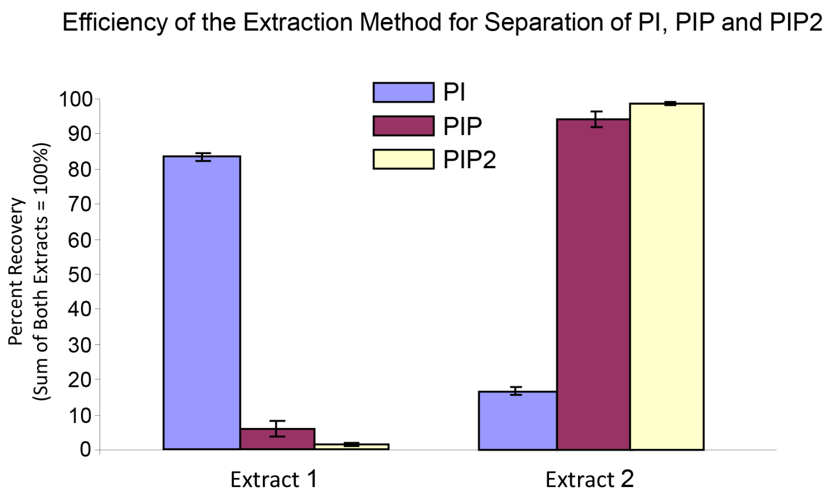

Supplementary Figure 8: Extraction of phosphatidylinositol (PI) and its phosphates phosphatidylinositol 4-phosphate (PIP) and phosphatidylinositol 4,5-bisphosphate (PIP2) from SKOV3 cells. A two-step approach adapted from the literature [2] was used showing excellent separation efficiency and reproducibility with a recovery of > 85% PI in 'Extract 1' and of > 95% PIP and PIP2 in 'Extract 2' (for technical details see Materials and methods).

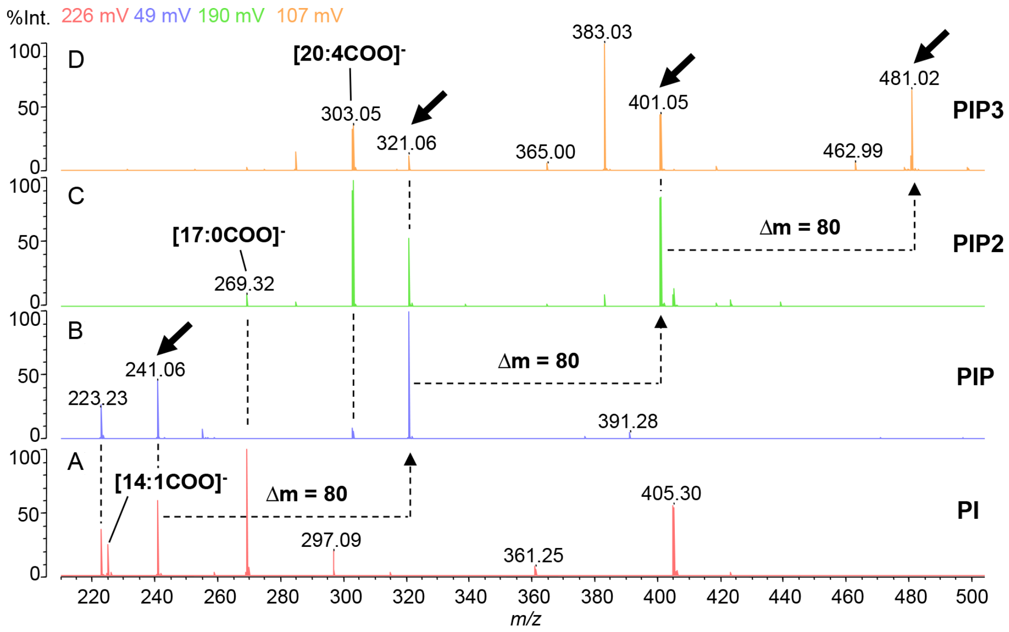

Supplementary Figure 9: Characteristic MALDI-MS/MS fragment ions. (**A**) phosphatidylinositol (PI) and its phosphates (**B**) phosphatidylinositol 4-phosphate (PIP), (**C**) phosphatidylinositol 4,5-bisphosphate (PIP2), and (**D**) phosphatidylinositol 3,4,5-trisphosphate (PIP3). Shown are the MS/MS spectra of (A) 1-heptadecanoyl-2-(9Z-tetradecenoyl)-sn-glycero-3-phospho-(1′-myo-inositol) (17:0–14:1 PI; Mw = 794.49), (B) 1-heptadecanoyl-2-(5Z,8Z,11Z,14Z-eicosatetraenoyl)-sn-glycero-3-phospho-(1′-myo-inositol-3′-phosphate) (17:0–20:4 PI(3)P; Mw = 952.51), (C) 1-heptadecanoyl-2-(5Z,8Z,11Z,14Z-eicosatetraenoyl)-sn-glycero-3-phospho-(1′-myo-inositol-3′,5′-bisphosphate) (17:0–20 : 4 PI(3,5)P2; Mw = 1032.47), and D) 1-heptadecanoyl-2-(5Z,8Z,11Z,14Z-eicosatetraenoyl)-sn-glycero-3-phospho-(1′-myo-inositol-3′,4′,5′-trisphosphate) (17:0–20 : 4 PI(3,4,5)P3; Mw = 1112.44). Bold arrows indicate the inositol phosphate ions showing a characteristic mass difference of 80 Da dependent on the number of phosphate groups esterified to the inositol head group. Carboxylate anions ([RCOO]-) indicating the fatty acid composition of the molecules are shown. All lipid standards were purchased from Avanti Polar Lipids (Alabaster, AL).

Supplementary Table 1: MALDI-MS/MS fragment ion analysis of the fatty acid composition of individual phosphatidylinositols (PI) and their phosphates PIP and PIP2 in SKOV3 cells.
see Supplementary_Table_1
